# Detailed Morphological Changes of Foveoschisis in Patient with X-Linked Retinoschisis Detected by SD-OCT and Adaptive Optics Fundus Camera

**DOI:** 10.1155/2015/432782

**Published:** 2015-08-18

**Authors:** Keiichiro Akeo, Shuhei Kameya, Kiyoko Gocho, Daiki Kubota, Kunihiko Yamaki, Hiroshi Takahashi

**Affiliations:** ^1^Department of Ophthalmology, Nippon Medical School Chiba Hokusoh Hospital, 1715 Kamagari, Inzai, Chiba 270-1694, Japan; ^2^Department of Ophthalmology, Nippon Medical School, 1-1-5 Sendagi, Bunkyo-ku, Tokyo 113-8602, Japan

## Abstract

*Purpose*. To report the morphological and functional changes associated with a regression of foveoschisis in a patient with X-linked retinoschisis (XLRS). *Methods*. A 42-year-old man with XLRS underwent genetic analysis and detailed ophthalmic examinations. Functional assessments included best-corrected visual acuity (BCVA), full-field electroretinograms (ERGs), and multifocal ERGs (mfERGs). Morphological assessments included fundus photography, spectral-domain optical coherence tomography (SD-OCT), and adaptive optics (AO) fundus imaging. After the baseline clinical data were obtained, topical dorzolamide was applied to the patient. The patient was followed for 24 months. *Results*. A reported *RS1* gene mutation was found (P203L) in the patient. At the baseline, his decimal BCVA was 0.15 in the right and 0.3 in the left eye. Fundus photographs showed bilateral spoke wheel-appearing maculopathy. SD-OCT confirmed the foveoschisis in the left eye. The AO images of the left eye showed spoke wheel retinal folds, and the folds were thinner than those in fundus photographs. During the follow-up period, the foveal thickness in the SD-OCT images and the number of retinal folds in the AO images were reduced. *Conclusions*. We have presented the detailed morphological changes of foveoschisis in a patient with XLRS detected by SD-OCT and AO fundus camera. However, the findings do not indicate whether the changes were influenced by topical dorzolamide or the natural history.

## 1. Introduction

X-linked juvenile retinoschisis (XLRS) is the most common inherited retinal dystrophy in males with an estimated prevalence at 1 : 5,000 to 1 : 20,000 [1, 2]. Mutations of the retinoschisis (*RS1*) gene are responsible for this disease [3]. XLRS is characterized by the presence of foveomacular cavities in the inner retina and a spoke wheel pattern of the retinoschisis in the macular area [2]. Recent reports suggest that carbonic anhydrase inhibitors (CAIs) administered topically or systemically can alleviate the maculopathy and improve the vision in approximately two-thirds of the patients [4–7].

Spectral-domain optical coherence tomography (SD-OCT) and adaptive optics (AO) fundus photography can obtain high-resolution images of diseased eyes that allow the detection of the early morphological changes of the retina.

We examined a man who was diagnosed with XLRS and followed the changes accompanying the reduction of his foveoschisis by both the SD-OCT and the AO fundus photography.

## 2. Methods

The protocol of this study conformed to the tenets of the Declaration of Helsinki and was approved by the Institutional Review Board of the Nippon Medical School. A signed written informed consent was obtained from patient after the nature and possible consequences of the study were explained. We had only one XLRS patient with foveoschisis to examine after the IRB approval. The patient was treated with topical dorzolamide, and it is still continued.

Blood samples were collected from the patient, and genomic DNA was isolated from the peripheral white blood cells using a blood DNA isolation kit (NucleoSpin Blood XL, Macherey Nagel, Germany). The DNA was used as the template to amplify the* RS1* gene. The coding regions and flanking introns of the* RS1* gene were amplified by polymerase chain reaction (PCR) using published primers [3] synthesized by Greiner Bio-One (Tokyo, Japan). The PCR products were purified (ExoSAP-IT,; USB Corp., USA) and were used as the template for sequencing. Both strands were sequenced on an automated sequencer (Bio Matrix Research, Chiba, Japan).

The ophthalmological examinations included measurements of the best-corrected visual acuity (BCVA), measurements of the refractive error, slit-lamp biomicroscopy, ophthalmoscopy, full-field electroretinograms (ERGs), multifocal ERGs (mfERGs), perimetry, fundus photography, fundus autofluorescence (FAF) imaging, SD-OCT, and AO imaging. Full-field scotopic and photopic ERGs were recorded using an extended testing protocol incorporating the International Society for Clinical Electrophysiology of Vision standards. The ERGs were elicited and recorded with a LED built-in electrode (LE2000, Tomey, Japan) [8]. The mfERGs were recorded using a commercial mfERG system (VERIS Science, Electro-Diagnostic Imaging, Inc., Redwood City, CA, USA) [9, 10]. The visual fields were determined by Goldmann perimetry. The FAF images were acquired with the TRC-NW8Fplus (TOPCON, Tokyo, Japan), and the SD-OCT images were acquired with a Cirrus HD-OCT (Carl Zeiss Meditec). The foveal thickness in the SD-OCT images was determined by a built-in measurement software. High-resolution fundus images were taken with the infrared AO retinal camera (rtx1, Imagine Eyes, Orsay, France). A detailed description on the use of this system to obtain images of individual cone photoreceptors was presented in detail earlier [11–16]. Briefly, the AO fundus camera illuminates a 4-degree square field with 850 nm infrared flashes to acquire* en face* images of the retina with a transverse optical resolution of 250 line pairs/mm. Successive AO images were taken at adjacent retinal locations with an angular overlap of 2 degrees in the horizontal and vertical directions. The resulting images were stitched together by superimposing retinal vessel landmarks with an image editing software (Photoshop, Adobe Corporation, Mountain View, CA; GIMP, The GIMP Development Team; ImageJ, National Institute of Health, Bethesda, MD). The pixel size of the images was typically 0.8 *μ*m when calculated at the retinal plane, and the value was adjusted for individual variations in the axial length of the eye [17]. The number and the mean width of retinal folds in AO were obtained at 500 *μ*m from the foveal center. The mean width of radial white line in the spoke wheel pattern in fundus photographs was also measured at 500 *μ*m from the foveal center.

## 3. Results 

The patient was a 42-year-old man who was initially diagnosed with XLRS in another hospital at the age of 30 years. Mutation analysis of the* RS1* gene found a missense mutation, c.608 C>T, in exon 6 with a substitution of leucine for proline at amino acid 203. This mutation has been reported in earlier reports on patients with XLRS [18, 19]. His older brother was also diagnosed with XLRS with the same mutation, and he had bilateral central atrophy without foveoschisis in the fundus photographs and SD-OCT images.

**Figure 1:**
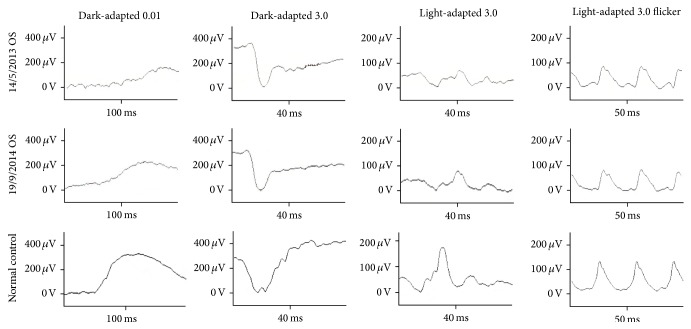
Time course of the changes in the full-field ERGs in the left eye of a patient with XLRS and a normal control is shown. The baseline of full-field ERGs recorded from the left eye of the patient and the same eye after 14 months of follow-up is shown on the top and middle row. The full-field ERGs of the normal control are shown in the bottom row. The dark-adapted 0.01, dark-adapted 3.0, light-adapted 3.0, and light-adapted 3.0 flicker ERGs of the full-field ERGs are shown. The amplitudes and implicit times of the full-field ERGs of the patient during the follow-up do not differ from those at the baseline.

**Figure 2:**
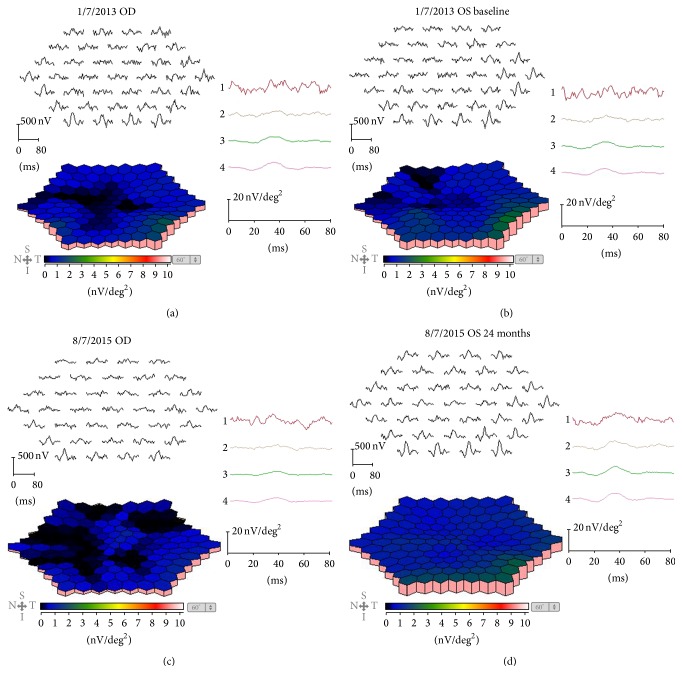
Local responses, topographic map, and average densities of the rings of the multifocal ERGs are shown. The baseline data from the right eye (a) and the left eye (b) of the patient and the data after 14 months of follow-up (c and d) are shown. The amplitudes of the mfERGs in the foveal area are severely reduced in both eyes at baseline. The amplitude of mfERGs after 14 months of follow-up is not changed.

The decimal best-corrected visual acuity (BCVA) of our patient was 0.15 in the right eye and 0.3 in the left eye. The intraocular pressure and anterior ocular segments were normal in both eyes. The amplitudes of both the cone and the rod full-field ERGs were reduced, and the waveforms were similar in both eyes. The dark-adapted 3.0 b wave of the ERG had a negative-type pattern in both eyes (Figure 1). The amplitudes of the mfERGs were reduced in the fovea and also in the peripheral areas in both eyes (Figure 2). Goldmann visual field examination showed the presence of central scotomas in both eyes.

**Figure 3:**
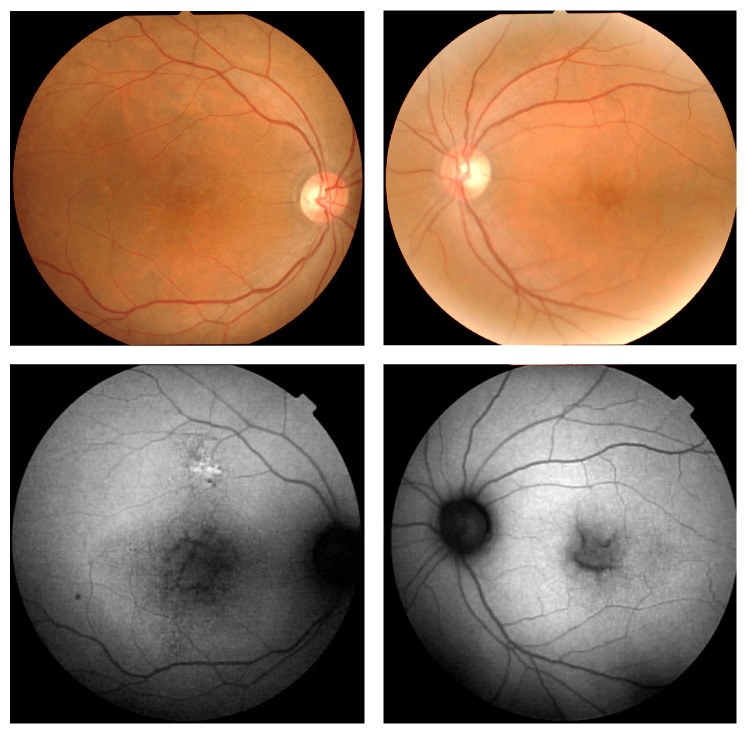
Fundus photographs and fundus autofluorescence (FAF) images of a patient with X-linked retinoschisis (XLRS). Fundus photographs show spoke wheel-like maculopathy in the left eye and central atrophy in the right eye. The FAF images show hypofluorescent areas in both maculas.

**Figure 4:**
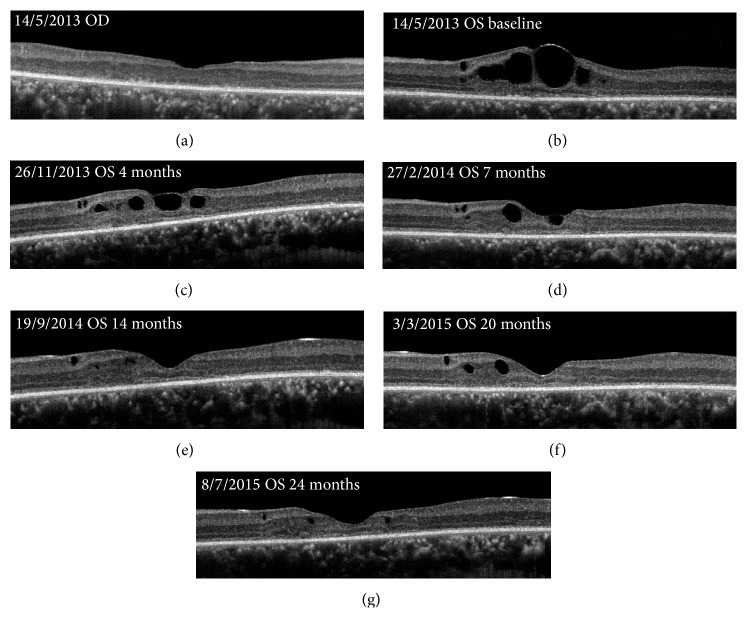
Time course of foveoschisis in SD-OCT images from the baseline is shown. (a) Baseline OCT image (180°) of the right eye showing central atrophy and a thinning of the entire retina. (b) Baseline OCT image (180°) of the left eye showing foveoschisis mainly in inner nuclear layer. The ellipsoid and interdigitation zones are not visible in the fovea of both eyes. (c) OCT image of the left eye after 4 months of treatment with dorzolamide. (d) OCT image of the left eye 7 months later. (e) OCT image of the left eye 14 months later. (f) OCT image of the left eye 20 months later. (g) OCT image of the left eye 24 months later.

**Figure 5:**
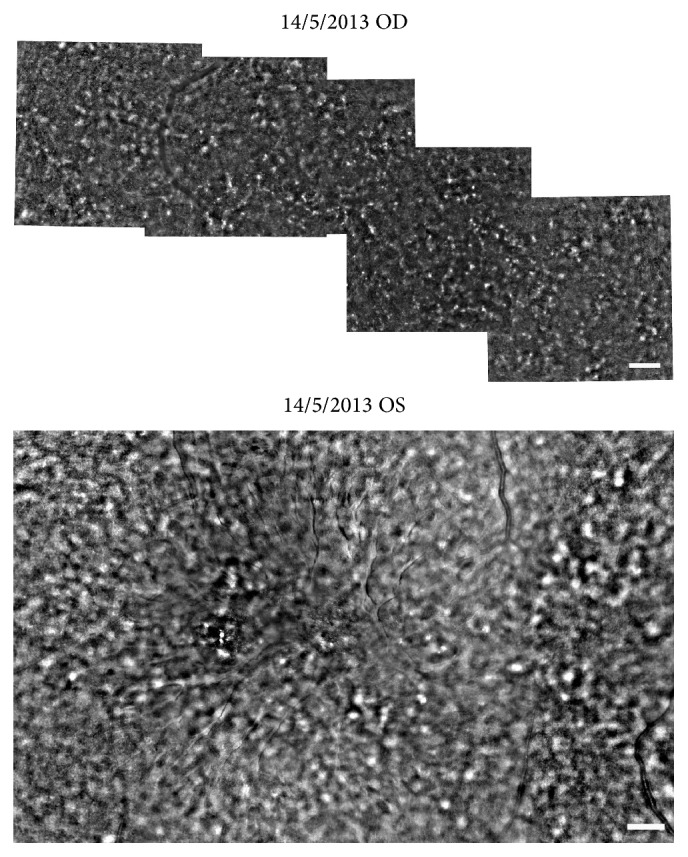
The baseline montage of AO images obtained from the macular region of the patient is shown. A montage of AO image of left eye shows spoke wheel pattern of the retinal folds in the inner retinal layer. The right eye does not show any folds. Bars 200 *μ*m.

Fundus examination showed spoke wheel-like maculopathy in the left eye and central atrophy in the right eye (Figure 3). The mean width of radial white lines in the spoke wheel pattern in the fundus photographs was 109 ± 21 *μ*m at 500 *μ*m from the foveal center in his left eye. Peripheral retinoschisis was not observed in both eyes. FAF imaging showed a hypofluorescent lesion in the macula of both eyes (Figure 3). The SD-OCT images showed a thinning of the total retinal thickness in right eye and foveoschisis mainly at the inner nuclear layer of the left eye. The ellipsoid and interdigitation zones were not detected in the fovea of the SD-OCT images of both eyes. The structure of inner nuclear layer, inner plexiform layer, and ganglion cell layer in the right eye was relatively preserved (Figure 4). Examination of a montage of the AO images of the left eye showed a spoke wheel pattern of retinal folds which had a thin inner retinal cavity possibly caused by the foveoschisis in the inner retinal layer (Figure 5). These folds were not present in the right eye (Figure 5). The width of spoke wheel retinal folds in the AO image in the left eye was thinner than that in the fundus photographs (Figures 3 and 5, Table 1). The cone mosaic was not clearly resolved throughout the retinal region, in which the ellipsoid and interdigitation zones were undetectable in SD-OCT images.

The left eye of the patient was treated with topical 2% dorzolamide three times/day, and the time course of the changes in the BCVA from the baseline is shown in Table 1. The amplitude of full-field (14 months from the baseline) and mfERGs (24 months from the baseline) was not changed during the posttreatment times (Figures 1 and 2).

**Figure 6:**
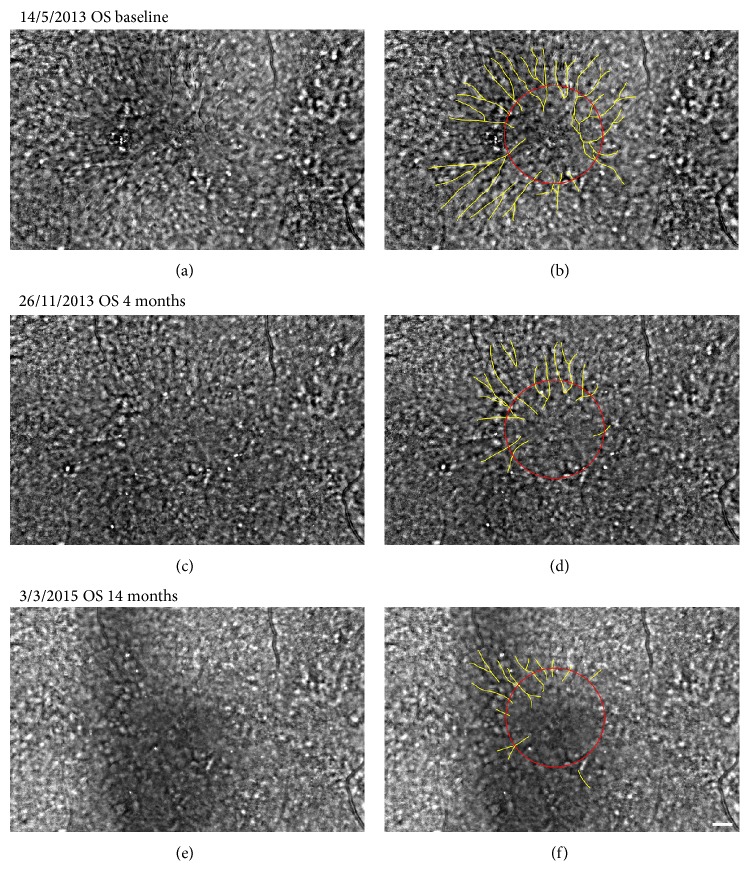
Time course of AO images and images (a, c, e) with highlighted retinal folds (b, d, f). (a) and (b) Baseline AO image (a) and image with highlighted retinal folds (b) of the left eye are shown. Spoke wheel pattern of retinal folds is highlighted by yellow line in (b). Red circle indicates 500 *μ*m circle from the fixation point. (c) and (d) AO image of the same eye after 4 months of treatment with dorzolamide is shown. (e) and (f) AO image of the same eye after 20 months of treatment with dorzolamide is shown. Bar 200 *μ*m.

Morphologically, cystoid macular edema in his left fundus became undetectable at the center of the fovea in 14 months from the baseline. The time course of the SD-OCT images showed a reduction of the retinal fluid and the foveal thickness in the left eye (Figure 4, Table 1). The number of the retinal folds in the AO images of his left eye was fewer than that at the baseline during the follow-up period (Figure 6, Table 1).

## 4. Discussion

In our patient with XLRS, we observed an improvement of the foveoschisis in the OCT images during the follow-up period, but the BCVA and the ERGs at the final visit were not better than those at the baseline. Topical and oral forms of CAIs have been demonstrated to also improve the BCVA and the macular cyst-like cavities in some cases of XLRS as documented by OCT [4–7]. We should be careful to interpret these results because Lesch et al. reported that the characteristics of the disease change from the schisis to the atrophic pattern after the second decade of the life [20]. It has also been shown that foveal thickness can fluctuate over time and these changes are not correlated with the functional changes [21]. The foveal photoreceptors might have already been potentially impaired by the chronic edema in our patient. This is supported by the disruption of the ellipsoid and interdigitation zones observed in the SD-OCT image at the baseline.

We should also be careful when we use dorzolamide for XLRS patients because Genead et al. stated that patients with XLRS who are receiving dorzolamide should be monitored for a potential rebound of their macular cysts or a lack of response during treatment [7].

This is the first study to show a spoke wheel pattern of the foveoschisis observed in the AO images. The regression of the number of the folds in the AO images corresponded to the regression of the foveoschisis observed by OCT. This observation supports our conclusion that the retinal folds observed in the AO fundus images were caused by foveoschisis.

## 5. Conclusions 

We have presented the high-resolution morphological changes of foveoschisis in a XLRS patient detected by SD-OCT and AO fundus camera. Unfortunately, we are not able to conclude whether the changes were influenced by dorzolamide or the natural history of the disease process because this is only one case. Both high-resolution morphological and functional analyses of a larger number of XLRS patients are needed to understand the relationship between the microstructural and functional effects of foveoschisis in retina. Further studies should help in determining this relationship.

## Figures and Tables

**Table 1:** Time course of the clinical data.

		BCVA^#1^	Foveal thickness in SD-OCT	Number of folds in AO^#2^	Mean width of folds in AO^#2^
		(OD/OS)	(*μ*m)		(*μ*m)
Pre treatment	2013.5	0.15/0.3	296	19	23.6 ± 8.0^#3^
Treatment started	2013.7				
Post treatment	2013.11	0.15/0.5	175	12	28.7 ± 12.9^#3,4^
	2014.9	0.1/0.3	86	—	—
	2015.3	0.1/0.4	80	8	27.0 ± 8.3^#3,4^
	2015.7	0.1/0.3	76	—	—

^#1^Best-corrected visual acuity.

^#2^Number and mean width of folds in AO are obtained at 500 *μ*m from the foveal center.

^#3^Mean ± standard deviation.

^#4^No significant difference compared with baseline (paired *t*-test).
